# Key insights from studies on the stability of personality disorders in different age groups

**DOI:** 10.3389/fpsyt.2023.1109336

**Published:** 2023-06-15

**Authors:** Delfine d’Huart, Süheyla Seker, David Bürgin, Marc Birkhölzer, Cyril Boonmann, Marc Schmid, Klaus Schmeck, Bo Bach

**Affiliations:** ^1^Department of Child and Adolescent Psychiatric Research, Psychiatric University Hospitals Basel, Basel, Switzerland; ^2^Department of Child and Adolescent Psychiatry and Psychotherapy, Ulm University, Ulm, Germany; ^3^Department of Forensic Child and Adolescent Psychiatry, University Psychiatric Clinics Basel, Basel, Switzerland; ^4^LUMC Curium—Department of Child and Adolescent Psychiatry, Leiden University Medical Center, Leiden, Netherlands; ^5^Center of Excellence on Personality Disorder, Psychiatric Research Unit, Region Zealand, Slagelse Psychiatric Hospital, Slagelse, Denmark

**Keywords:** personality disorders, personality disorders symptoms, mean-level stability, rank-order stability, review

## Abstract

While for decades, temporal stability has been conceived as a defining feature of personality disorders (PDs), cumulative findings appear to question the stability of PDs and PD symptoms over time. However, stability itself is a complex notion and findings are highly heterogenous. Building upon a literature search from a systematic review and meta-analysis, this narrative review aims to capture key findings in order to provide critical implications, both for clinical practice and future research. Taken together, this narrative review revealed that unlike previous assumptions, stability estimates in adolescence are comparable to stability estimates in adulthood and PDs and PD symptoms are not that stable. The extent of stability itself depends yet on various conceptual, methodological, environmental, and genetic factors. While findings were thus highly heterogenous, they all seem to converge in a notable trend towards symptomatic remission, except for high-risk-samples. This challenges the current understanding of PDs in terms of disorders and symptoms and argues instead in favor of the AMPD and ICD-11 reintroducing the idea of self and interpersonal functioning as the core feature of PDs.

## 1. Introduction

Traditionally conceived as a defining feature of personality disorders (PDs), stability has quickly become a major concern, adding to the ongoing debate about the procedure of conceptualizing and diagnosing a PD. For decades, temporal stability has been a major factor in distinguishing axis I from axis II disorders with the stability of PDs being considered to be higher than for other mental disorders. Cumulative findings, however, gradually challenged the stability of PDs, indicating a notable trend towards improvement over time (1, 2). Unlike previous assumptions, PDs have thus not been found to be much more stable than other mental disorders (3). Nevertheless, stability is a complex notion that should be assessed in the light of several factors (4, 5). As such, PDs may be conceptualized in multiple ways including categories, symptom counts, and pathological traits. Similarly, various conceptually and statistically distinct approaches may lead to distinct types of stability. These different types, then, may depend on various methodological factors, such as sampling procedures (i.e., age range, clinical status, follow-up interval), the assessment modality, and the type of instrument being used. As a result, study findings are highly heterogenous, and misconceptions about the course of PDs still seem to remain.

In this narrative review, we capture key findings of the current literature on the stability of PDs across different age groups and critically discuss general implications for both clinical practice and future research. We start by describing different PD constructs and different types of stability, followed by an overview of recent studies in childhood, adolescence, and adulthood. Finally, we emphasize key findings and conclude with general implications.

## 2. Personality disorder constructs

PDs can be conceptualized according to different constructs, features, and frameworks. As such, in both the fifth edition of the Diagnostic and Statistical Manual of Mental Disorders [DSM-5 (6)]; and the 10th edition of the International Classification of Disorders [ICD-10 (7)], PDs are defined as discrete categories, each with a distinct set of diagnostic criteria (i.e., either a PD is present or not). Within this categorical system, PDs can also be conceptualized more dimensionally, in terms of symptom counts (e.g., seven out of nine borderline PD symptoms). In recent PD models, such as the Alternative Model of Personality Disorders (AMPD) in section III of the DSM-5 (6), as well as the 11th edition of the ICD (7), PDs are, moreover, perceived in terms of core impairments in personality functioning (i.e., self-, and interpersonal functioning), specified by a set of pathological traits (i.e., extreme variants of normal personality dimensions, such as emotional lability, attention seeking or impulsivity). These different constructs and approaches may naturally affect stability estimates. Although a growing number of longitudinal studies investigate dimensional measures of personality pathology [e.g. (8, 9)], previous research has focused primarily on PD categories and PD symptoms counts, except for child-and adolescent studies focusing exclusively on maladaptive personality traits. Therefore, the current review focusses exclusively on DSM and ICD based categorical and symptom-based models.

## 3. Different types of stability

Apart from the aforementioned constructs, multiple ways to describe stability over time are common, and stability itself tends to differ according to the type of stability assessed. In the present review, we focus on the two types of stability that have been studied most frequently, namely, mean-level and rank-order stability.

Mean-level stability refers to the degree to which the average level of a PD or PD symptom changes over time. Categorical mean-level stability, also known as diagnostic stability, then refers to the consistency of PD diagnoses, typically measured through the proportion of enduring cases from baseline to follow-up (e.g., four out of ten participants, who were diagnosed with BPD at baseline, still meet the criteria at follow-up, resulting in a categorical mean-level stability of 40%). Dimensional mean-level stability then refers to the consistency of PD symptom counts, usually measured by mean-difference scores (i.e., difference between mean symptom count at follow-up and mean symptom count at baseline).

Rank-order stability, in turn, refers to the consistency of an individual’s relative ordering compared to others in a given sample, indicating thus the degree to which interindividual differences are preserved over time. As such, individuals may retain their relative ordering with regard to a specific PD or PD symptom over time, even if the average level of a PD or PD symptom in a given sample increases or decreases over time. Subsequently, rank-order changes are independent of mean-level changes (10). Categorical rank-order stability then refers to the rank-order stability of individuals’ PD diagnosis, typically measured with Cohen’s κ. While a negative value indicates no agreement, a κ between 0 and 0.20 indicates a low, a κ between 0.21 and 0.40 a fair, and a *κ* between 0.41 and 0.60 a moderate agreement. A *κ* between 0.61 and 0.80, then, indicates a substantial agreement, and a *κ* between 0.81 and 1.0 a perfect agreement (11). Dimensional rank-order stability, in turn, refers to the rank-order stability of an individuals’ PD symptom count, commonly measured through a test-retest correlation (e.g., Pearson’s *r*). A *r* between 0.1 and 0.3 is said to be low, a *r* between 0.3 and 0.5 moderate, and a *r* between 0.5 and 0.8 high (12). Another powerful method to assess the stability of PDs over time, consists in using structural equation models. Structural equation models encompass a set of multivariate approaches [e.g., individual growth curve models (13); growth mixture modeling (14)] that allow to distinguish between measurement error and true individual differences related to change processes.

## 4. Overview of the current literature review

The literature search for this narrative review was part of a systematic review and meta-analysis, *conducted in accordance with the PRISMA standards* (15) *as well as the MOOSE guidelines* (16). The literature search conducted in four electronic databases (EMBASE, PsycInfo, PubMed, and Web of Science) on October 26, 2020, and updated on June 7, 2022 (d’Huart et al., under review). Keywords and Medical Subject Headings (MeSH) terms were used to identify peer-reviewed articles reporting on the stability of PDs between 1980 and 2022. In brief, following search terms were used in the literature search: “personality disorders,” “axis II disorders,” “stability,” “consistency,” “longitudinal,” “prospective,” “life span,” and “life course.” Only longitudinal studies, assessing the stability of PDs at two different time points at least 1 month apart, were considered for the current paper. Studies will be presented from a developmental perspective, including childhood, adolescence, and adulthood. A complete overview is given in Tables 1–3.

**Table 1:** Longitudinal studies on the course of PDs in childhood (*k* = 2).

Author(s) and publication year	Sample size^a^	Time interval^c^	Mean age^b^ *M* (SD)	Setting	Assessment of PDs and PD traits				Main outcome
					PD construct	Type of stability	Type of PD	Instrument	
Crick et al., 2005 (17)	400	24	NR	Clinical	Traits	Rank-order (D)	BPD	BPFS-C	Moderate dimensional rank-order stability
de Clercq et al., 2009 (18)	477	12	10.67	Clinical	Traits	Mean-level; Rank-order (D)	BPD	DIPSI	The children’s maladaptive trait scores generally decreased as they grow older; substantial dimensional rank-order stability

BPD, borderline PD; BPFS-C, borderline personality features scale for children; DIPSI, dimensional personality symptom item pool; NR, not reported.

a Sample size used for the analyses.

b Mean age at baseline.

c The follow-up interval is displayed in months.

**Table 2:** Longitudinal studies of the course of PDs in adolescence (*k* = 10).

Author(s) and publication year	Sample size^a^	Time interval^c^	Mean age^b^ *M* (SD)	Setting	Assessment of PDs and PD traits				Main outcome
					PD Construct	Type of stability	Type of PD	Instrument	
Bernstein et al., 1993 (19)	733	24	16.30 (2.8)	Community	Categories	Diagnostic	Any PD	SCID-II	Most PD diagnoses did not persist over time; subjects with PDs identified earlier remained at elevated risk for receiving a PD again at follow-up
Bornovalovaet al., 2009 (20)	1,118	120	NR	Community	Symptoms	Mean-level	BPD	MPQ-BPD	Significant mean-level decline from age 14 to 24
Chanen et al., 2004 (21)	96	24	16.10 (0.9)	Clinical	Categories; symptoms	Diagnostic; rank-order (C, D) mean-level	DSM-5 PDs	SCID-II	74% retained any PD diagnosis over time; low to high cat and dim rank-order; low to high mean-level stability
d’Huart et al., 2022 (10)	115	120	15	CW & JJS	Categories; symptoms	Diagnostic; rank-order (C, D) mean-level	DSM-5 PDs	SCID-II	47% retained the diagnoses over time; significant increases of small to moderate effect sizes; moderate cat rank-order stability; low to moderate dimensional rank-order stability
Greenfield et al., 2015 (22)	204	48	14.6 (1.5)	Clinical	Categories	Diagnostic	BPD	Ab-DIB	76% retained the diagnosis over time
Grilo et al., 2001 (23)	60	24	15.60 (1.7)	Clinical	Symptoms	Mean-level	DSM-5 PDs	PDE	Significant declines for histrionic, narcissistic, dependent, obsessive-compulsive, and passive-aggressive PDs; low to moderate mean-level stability
Hamlat et al., 2020 (24)	675	36	11.60 (2.4)	Community	Symptoms	Mean-level; rank-order (D)	STPD; HPD; BPD; APD; DPD	APD	Significant declines of small to medium effect sizes; moderate to high dimensional rank-order stability
Johnson et al., 2000 (25)	816	108	13.80 (2.57)	Community	Symptoms	Mean-level; rank-order (D)	DSM-5 PDs	DISC-I	PD symptoms were highest in adolescence and declined linearly to adulthood, although effect sizes were small; low to moderate dimensional rank-order stability; cluster C symptoms seemed to be less stable than cluster A and B symptoms
Strandholm et al., 2017 (26)	189	12	16.40 (1.61)	Clinical	Symptoms	Mean-level; rank-order (D)	DSM-5 PDs	SCID-II	Significant declines for most of PD symptoms; low to moderate cat rank-order stability
Yen et al., 2013 (27)	99	6	15.3	Clinical	Symptoms	Rank-order (C)	BPD	CI-BPD	Low cat rank-order stability

CW & JJS, child welfare and juvenile justice sample; STPD, schizotypal PD; HPD, histrionic PD; BPD, borderline PD; APD, avoidant PD; DPD, dependent PD; SCID-II, structured clinical interview for DSM-IV personality sisorders; MPQ-BPD, multidimensional personality questionnaire-borderline personality disorder scale; PDE, personality disorder examination; Ab-DIB, abbreviated diagnostic interview of borderlines; DISC-I, diagnostic interview schedule for children; NR, not reported.

a Sample size used for the analyses.

b Mean age at baseline.

c The follow-up interval is displayed in months.

**Table 3:** Longitudinal studies on the course of PDs in adulthood (*k* = 28).

Author(s) and publication year	Sample size^a^	Time interval^c^	Mean age^b^ *M* (SD)	Setting	Assessment of PDs and PD symptoms				Main outcome
					PD construct	Type of stability	Type of PD	Instrument	
Alvarez-Tomàs et al., 2017 (28)	41	120	26.90 (6.3)	Clinical	Categories; symptoms	Diagnostic; mean-level	BPD	SCID-II	50% of participants retained their diagnosis over time; significant decreases in BPD symptoms
Black et al. (1995) (29)	26	540	NR	Clinical	Category	Diagnostic	ASPD	DIS	58% showed (complete) remission 42% showed no remission
Bukh et al., 2017 (30)	262	69.6	NR	Clinical	Categories	Diagnostic	Any PD	SCID-II	72% retained a PD over time
Conway et al., 2018 (31)	1,630	60	59.6	Community	Symptoms	Rank-order (D)	BPD	SIDP	High dimensional rank-order stability over time
de Groot et al., 2003 (32)	72	72	NR	Clinical	Symptoms	Rank-order (D)	DSM-5 PDs	MCMI-II	Significant changes for some PD symptoms, whereas others were found to be highly stable
Durbin and Klein, 2006 (33)	101	120	32.0 (9.6)	Clinical	Symptoms	Diagnostic; mean-level; rank-order (D)	DSM-5 PDs	PDE	Poor to fair categorical mean-level stability; fair to moderate dimensional mean-level stability; growth curve analyses revealed, however, complex patterns of change in mean scores of PD symptoms
Farabaugh et al., 2007 (34)	129	6.5	42.5 (8.91)	Clinical	Categories	Diagnostic	Any PD; BPD	SCID-II	50% of the participants retained their PD diagnosis over time
Hopwood et al. 2013 (35)	266	120	NR	Clinical	Symptoms	Rank-order (D)	DSM-5 PDs	DIPD-IV	Self-reported PD symptoms were substantially higher than clinical interviews PD symptoms both before and after correcting for retest dependability and internal consistency values
Kullgren and Armelius, 1990 (36)	41	60	30.90 (7.3)	Clinical	Categories	Diagnostic	BPD	DIB	Diagnostic stability was low and only 56% of all patients retained their diagnosis on follow-up
Laptook et al., 2006 (37)	127	120	31.4	Clinical	Symptoms	Rank-order (C)	DPD	SCID-II	The cat rank-order stability of the diagnosis was fair to moderate
Lenzenweger et al., 1999 (38)	250	48	NR	Community	Symptoms	Rank-order (D)	DSM-5 PDs	IPDE	Significant modest declines in PD symptoms over time, while effect sizes were small; high dim rank-order stability
Lopez-Castroman et al., 2012 (39)	82	3	38.60 (11.6)	Clinical	Categories	Diagnostic	Any PD	SCID-II	80% retained the diagnosis over time
Loranger et al., 1991 (40)	84	6	29.70 (8.7)	Clinical	Categories; symptoms	Diagnostic; rank-order (C); mean-level	Any PD; DSM-5 PDs	PDE	73% retained the diagnosis of any PD over time. Notable trend towards fewer symptoms at follow-up than at baseline. Moderate cat rank-order stability
Mulder et al., 2010 (41)	149	18	31.6 (NR)	Clinical	Categories; symptoms	Diagnostic; mean-level	Any PD; DSM-5 PDs	SCID-II	52% retained the PD diagnosis over time; low to moderate diagnostic stability; significant decreases in PD symptoms over time
Nestadt et al., 2010 (42)	294	180	47.00 (NR)	Community	Categories; symptoms	Diagnostic; mean-level	DSM-5 PDs	PDS	OCPD exhibited substantial mean-level stability; ASPD, APD, BPD, HPD, STPD exhibited moderate mean-level stability; DPD, NPD, PPD, SPD exhibited low mean-level stability
Nysaeter et al., 2012 (43)	14	24	28.90 (6.1)	Clinical	Categories	Diagnostic	BPD	SCID-II	32% retained the diagnosis over time
Paris and Zweig-Frank (2001) (44)	64	324	50.00	Clinical	Categories; symptoms	Diagnostic; mean-level	BPD	DIB	7.8% retained the diagnosis over time. Significant decreases in BPD symptoms over time
Reichborn-Kjennerud et al., 2015 (45)	2′282	115.2	28.20 (NR)	Community	Categories; symptoms	Diagnostic; rank-order (D)	ASPD; BPD	SCID-II	General declines for both disorders; moderate (BPD) to high (ASPD) dim rank-order stability
Riihimäki et al., 2014 (46)	111	60	37.30 (13.7)	Clinical	Categories	Diagnostic	BPD	SCID-II	57% patients in depressive primary care retained a BPD diagnosis over 5 years
Schilders et al., 2017 (47)	776	52.8	NR	Community and prison	Categories	Diagnostic	Any PD	DIB	30% across settings retained the diagnosis over time; diagnostic stability was higher in prison than in the community setting
Silk et al., 1990 (48)	9	27	NR	Clinical	Categories	Diagnostic	BPD	DIB	56% patients retained a BPD diagnosis over time
Trull and Goodwin, 1993 (49)	44	6	28.59 (8.12)	Clinical	Symptoms	Mean-level	DMS-5 PDs	SCID-II	Significant decreases in PD symptoms over time
Vaglum et al., 1993 (50)	73	33.6	35.00 (9.00)	Clinical	Categories	Diagnostic; rank-order (C)	Any PD	SCID-II	56% retained a PD diagnosis at follow-up; high cat rank-order stability
Vater et al., 2014 (51)	40	24	30.18 (6.98)	Clinical	Symptoms	Mean-level	NPD	SCID-II	NPD symptoms significantly decreased across time
Vergara-Moragues et al., 2013 (52)	200	3	35.01 (7.7)	Clinical	Symptoms	Mean-level	PPD; SPD; STPD; HPD; NPD; ASPD; APD; DPD; OCPD	MCMI-II	Most of PD symptoms in psychoactive substance abuse patients had significantly decreased over time
Vrabel et al., 2010 (53)	74	60	29.40 (7.3)	Clinical	Categories	Diagnostic	Any PD	SCID-II	55% of the patients with longstanding eating disorders retained their initial diagnosis; significant decreases of PD symptoms over time
Zanarini et al., 2010 (54)	247	120	26.90 (5.8)	Clinical	Categories	Diagnostic	BPD	R-DIB	50% of BPD participants achieved a recovery over time

PBD, paranoid PD; SPD, schizoid PD; STPD, schizotypal PD; HPD, histrionic PD; NPD, narcissistic PD; BPD, borderline PD; ASPD, antisocial PD; APD, avoidant PD; DPD, dependent PD; OCPD, obsessive-compulsive PD; SCID-II, structured clinical interview for DSM-IV personality disorders; DIS, diagnostic interview schedule; SNAP, schedule for non-adaptive and adaptive personality; MCMI-II, millon clinical multiaxial inventory-II; PDE, personality disorder examination; DIPD-IV, diagnostic interview for DSM-IV personality disorders; DIB, diagnostic interview for borderlines; SIDP, structured interview for DMS-III-personality disorders; IPDE, international personality disorders examination; PDS, personality disorder schedule from the standardized psychiatric examination (SPE); BPD, borderline personality disorder; R-DIB, revised diagnostic interview for borderlines; NR, not reported.

a Sample size used for the analyses.

b Mean age at baseline.

c The follow-up interval is displayed in months.

### 4.1. Childhood

Only two studies to date, namely the studies from Crick et al. (17) and the study from de Clercq et al. (18), have examined the stability of maladaptive personality traits in childhood. While both studies exclusively focused on borderline PD (BPD) traits among community-based, primary school-aged children, they differed regarding the instrument type and the follow-up period, as described in Table 1. While Crick et al. (17) only investigated dimensional rank-order stability, de Clercq et al. (18) investigated both, dimensional rank-order and dimensional mean-level stability. Thus, Crick et al. (17) found only moderate dimensional rank-order stability, while de Clercq et al. (18) found substantial dimensional rank-order stability over time. de Clercq et al’s (18) findings on dimensional mean-level stability indicated that children’s maladaptive trait scores generally decreased as they grow older, with a smaller decline for children who initially had higher levels of maladaptive personality traits.

### 4.2. Adolescence

Overall, ten studies reported data on the stability from adolescence to adulthood (see Table 2). Five studies were from clinical settings (21–23, 26, 27), four studies from community-based samples (19, 20, 24, 25) and one study from a high-risk sample [i.e., young adults with a history of child welfare and juvenile justice placements (10)]. From the studies conducted in clinical settings, two studies (21, 23) were conducted among patients with mixed axis I comorbidities, two studies (22, 27) were conducted among previously suicidal youth and one study (26) was conducted among depressed adolescent outpatients. Three studies (20, 22, 27) focused exclusively on BPD, while the remaining seven studies focused on any PD or most of the DSM-5 PDs. The follow-up period ranged between 6 months (27) and 10 years (10, 20) and four studies (10, 19, 21, 26) used the Structured Clinical Interview for DSM-IV Personality Disorders (SCID-II), while the remaining six studies (20, 22–25, 27) each used different measurement instruments, as presented in Table 2. Most studies focused on PD symptom counts, with only four studies (10, 19, 21, 22) investigating PD categories, three studies (10, 21, 27) reporting data on categorical rank-order stability, seven studies (10, 20, 21, 23–26) reporting data on dimensional mean-level stability, and five studies (10, 21, 24–26) reporting data on dimensional rank-order stability. Findings on diagnostic stability included two studies (21, 22) suggesting substantial stability over time and two studies (10, 19) suggesting only moderate estimates over time. Findings on categorical rank-order stability included two studies (9, 14) indicating moderate categorical rank-order stability for any PD and low to high categorical rank-order stability for individual PD diagnoses, and one study (23) suggesting low categorical rank-order stability for a BPD diagnosis. Findings on dimensional mean-level stability, however, consistently indicated significant decreases for most of PD symptoms over time (20, 21, 23–26). Only one study (10), revealed significant increases for most of PD symptoms over time. The authors concluded that this finding may be explained by the nature of the high-risk sample, as many adolescents in the child welfare and juvenile justice system have experienced severe childhood adversities (e.g., child abuse and neglect) as well as a range of other critical risk factors (i.e., unfavorable parenting practices, low socioeconomic status, childhood psychopathology, self-harming behavior, and youth delinquency) which all have been shown to be significantly associated with the stability of PDs over time. Finally, findings on dimensional rank-order stability revealed highly heterogenous patterns, with three studies (10, 25, 26) ranging from low to moderate, one study (21) ranging from low to high, and one study (24) ranging from moderate to high, depending on PD types.

### 4.3. Adulthood

Overall, 28 studies investigated the stability of PDs in adulthood (see Table 3). Most studies were from clinical settings and only four studies were from community-based samples (29, 31, 38, 42, 44, 45). One study was based on a mixed sample, including both community-based and incarcerated adults (47). Among the studies in clinical settings, seven were conducted among depressed outpatients (30, 33, 34, 37, 39, 41, 48), two were conducted among substance abuse patients (32, 52), and one was conducted among adults with long-standing eating disorders (53). The remaining studies (28, 29, 35, 36, 40, 43, 44, 46, 49, 50) included patients with mixed axis I comorbidities. In addition, nine studies focused exclusively on BPD patients (28, 31, 36, 43, 44, 46, 48, 54), one study focused on BPD and antisocial PD (i.e., ASPD) (45), one study exclusively focused on ASPD (29), and study exclusively focused depressive PD [DPD (37)]; and one study exclusively focused on narcissistic PD (i.e., NPD) (51). The remaining studies either examined “any PD” (30, 34, 39, 50, 53) or DSM-5 PDs (32, 33, 35, 38, 42, 49, 52). The follow-up period varied between 3 months (39) and 45 years (29) and most studies used the SCID-II. In contrast to studies conducted among adolescents, most studies in adulthood focused on PD categories. Thus, 19 studies (28–30, 33, 34, 36, 39–48, 50, 55) reported data on diagnostic stability, revealing highly heterogenous findings, ranging from 7.8% (44) to 80% (39). Three studies (37, 40, 50) reported data on categorical rank-order stability, with two studies (28, 39) indicating moderate and one study (43) indicating high categorical rank-order stability. Nine studies (28, 33, 40–42, 44, 49, 51, 52) reported data on dimensional mean-level stability, consistently suggesting significant declines for most of PD symptoms over time. Finally, six studies (31–33, 35, 38, 45) reported data on dimensional rank-order stability, revealing findings ranging from low to high, depending on the specific type of PD being assessed.

## 5. Insights from the current literature review

Six key findings emerged from the current literature review, which warrant a more detailed discussion.

### 5.1. Stability estimates in adolescence are comparable to those in adulthood

Although research focusing on adolescence has substantially increased over recent years, the number of studies assessing the stability of PDs in childhood and adolescence still appears to be low when compared to studies in adulthood. Part of this may be due to the widespread reluctance to diagnose PDs in adolescence because of the stigma associated with the disorder (56, 57) and the belief that personality in adolescence itself is driven by strong emotions and impulsive behavior (58, 59). Yet recent literature clearly indicates that PDs can be validly and reliably diagnosed prior to the age of 18 years (58–60) and that the stability in adolescence is comparable to that in adulthood. Nevertheless, while maladaptive personality traits can be found as early as childhood, it is reasonable to assume that more severe forms of PDs only become clinically apparent in later adolescence, when individuals have acquired skills to integrate knowledge about themselves and others into a coherent self-identity (61).

### 5.2. Except for high-risk samples, most PD diagnoses and PD symptoms tend to decrease over time, regardless of age

Although most studies largely differed in terms of methodological and conceptual factors, they all seem to converge in the fact that most PD categories (i.e., diagnostic stability) and PD symptoms (i.e., dimensional mean-level stability) decrease over time, while individuals’ rank-ordering (i.e., dimensional rank-order stability) seems to persist. Specifically, studies on the diagnostic stability, overall revealed that many individuals diagnosed with a PD at baseline are likely to not fulfill diagnostic criteria at follow-up. This is most notable, highlighting one of the major shortcomings of the categorical PD system for specific PDs in being based on an arbitrary diagnostic threshold that can easily be met (diagnosis PD) or unmet (no diagnosis PD) by an increase or decrease in a single criterion. This, indeed, favors diagnostic instability, while minor changes in the pathology remain unidentified and the subclinical expression of the individual’s symptoms may remain high (62). Thus, the diagnostic stability of specific PDs appears to be a rather inappropriate measure to assess the stability of PDs over time, as a categorical scaling leads to a substantial loss of information. This shortcoming could be in part compounded by looking at the stability of any PD (including PD NOS) rather than the diagnostic stability of specific PDs. As such, it may be that patients change specific categorical diagnoses but fait to discard the general diagnosis of any PD. Studies on dimensional mean-level stability mostly suggested considerable declines of PD symptoms over time. Although one might think that this is mainly due to treatment effects (63) significant decreases were also found in community-based samples, which suggests a rather natural improvement. While in healthy personality research, mean trait levels tend to change toward increasing maturity in community based settings over time [i.e., decrease in neuroticism, increase in extraversion, agreeableness, and conscientiousness (64)], this might be true for PD traits too. Indeed, the findings of Wright et al. (65), showed that decreases in avoidant PD traits were associated with increases in dominance and warmth and decreases in neuroticism. Studies on dimensional rank-order stability, however, generally indicated moderate to high stability estimates, meaning that individuals who exhibited high levels of a specific PD symptom at one time point also showed relatively high levels of that symptom at a second time point. Taken together, the mean-level of PDs and PD symptoms tends to decrease over time, regardless of participants’ age. Participants’ rank-ordering, however, tends to persist.

### 5.3. Stability estimates tend to vary with respect to study-specific factors

The extent of stability, nonetheless, considerably differed across studies, depending on the PD construct (i.e., categorical diagnoses or dimensional symptoms), the type of stability (i.e., diagnostic, mean-level or rank-order stability), and the specific PD and PD symptom being assessed. In addition, studies differed largely with respect to methodological factors, which yet again, influenced stability estimates. As such, at least six different findings must be emphasized: (a) stability estimates tend to be considerably higher when PDs are assessed dimensionally (i.e., PD symptom counts or PD traits) compared to PDs assessed categorically (PD categories). For instance, the study from Durbin and Klein (33) suggested poor to fair stability estimates for PD categories, while the stability for dimensional PD symptoms were found to be fair to moderate; (b) dimensional rank-order stability estimates seem to be higher than dimensional mean-level stability estimates, meaning that PD symptoms tend to decrease on average, while individual’s rank-ordering in a given sample remains almost the same (33, 66); (c) dimensional stability estimates appear to be higher for self-reported PD symptoms than for interview-based PD symptoms (33, 35, 38, 67). As such, Lenzenweger (38) found smaller 4 years dimensional rank-order stability estimates for interview-based PD symptoms (*r* = 0.61) than for self-reported symptoms (*r* = 0.70). Consistently, Durbin and Klein’s (33) stability estimates were 0.49 for interview-assessed symptoms and 0.69 for self-reported symptoms; (d) shorter sampling intervals will generally result in higher stability estimates compared to longer sampling intervals. For instance, dimensional mean-level changes in the Collaborative Longitudinal Personality Disorders Study [CLPS (68)]; were described as “small” at a 2 years follow-up, “medium” at a 4 years follow-up, and “large” at a 10 years follow-up interval; (e) in terms to the type of PD being assessed, cluster B PDs seem to be generally more stable than cluster A and C PDs (25); (f) PD patients in clinical settings seem to attain symptomatic remission more quickly than those from community-based samples. According to Morey and Hopwood (4), one possible reason could be that in clinical samples, participants are often drawn from treatment settings, targeting clinical remission. Therefore, participants in clinical settings tend to show faster declines (i.e., lower stability) compared to other settings. In sum, the extent of stability considerably differs according to the PD type and construct, the type of stability being assessed and several methodological factors, such as the assessment modality, sampling interval, and clinical setting.

### 5.4. Stability estimates tend to vary with respect to environmental and genetic factors

In addition to conceptual and methodological factors, stability estimates, however, also seem to vary as a function of environmental and genetic factors. According to behavioral genetics research, individuals may be genetically predisposed to exhibit more or less stable personality traits. In other words, an individual’s overall score of PD symptoms as well as the extent to which this individual exhibits symptomatic change is strongly heritable (20). Yet individuals evolve within specific environments which may considerably affect stability estimates. As such, the study from Reichborn-Kjennerud and colleagues (45) indicated that the rank-order stability of ASPD and BPD symptoms was largely due to genetic factors, whereas symptomatic change was due to environmental risk factors. Bornovalova and colleagues (20), in contrast, found that stability and change in BPD symptoms were substantially affected by genetic factors and only modestly by environmental factors. However, the authors point out that the strong influence of genetic factors does not mean that environmental factors are unimportant, but rather indicate that the environment, indeed, is likely to influence gene expression, and emphasize the need for interventions to ensure that the individual’s family may serve as a protective factor against the manifestation of pathological traits.

### 5.5. Symptomatic remission does not equate full recovery

Although study findings overall suggest that most PD categories and PD symptoms decrease over the lifespan, it should be kept in mind that a symptomatic remission is not necessarily accompanied by full recovery. Thus, while symptomatic remission is defined as no longer meeting diagnostic criteria for at least 2 years, full recovery is defined as attaining good social and vocational functioning in addition to symptomatic remission. In the McLean Study of Adult Development [MSAD (69)], 34.6% of BPD patients had remitted by the time of the first follow-up (2 years after the baseline assessment), about half (49.5%) had remitted by 4 years follow-up, 69% at 6 years follow-up and 93% had remitted at a 10 years follow-up (2, 54, 70). By the time of the 16 years follow-up assessment, nearly all patients (99%) had experience symptomatic remission and symptom decline stayed relatively stable, with only few patients experiencing symptomatic recurrence (55). However, notably, only half of the patients had achieved significant functional improvements over the 16 years follow-up, with some even experiencing relapse or worsened functioning. Accordingly, the authors conclude that good social and vocational functioning is more difficult to attain than symptomatic remission and, therefore, sustained recovery is much less common than sustained symptomatic remission from BPD. A decrease in PD symptoms is thus not necessarily accompanied by an increase in social and vocational functioning.

### 5.6. Studies in high-risk samples are scarce

Finally, studies investigating the stability of PDs in high-risk samples are surprisingly scarce. Thus, only two studies (10, 47) examined stability estimates in high-risk samples, namely in adolescents placed in the child welfare and juvenile justice system (10) and incarcerated adults (47). This is especially striking given that individuals from high-risk samples are particularly at risk for developing a PD. Consistently, both studies (10, 47) suggested substantial increases in PD diagnoses over time (11, 45), while clinical and community-based studies overall converged in that most PD diagnoses and symptoms decrease over time.

## 6. Implications

Overall, studies suggest that PDs, either assessed categorically or dimensionally, are not as stable as previously assumed. This highlights the need to overcome the clinical assumption that PDs are “enduring,” “pervasive” and “inflexible” over time. This emphasizes that PDs are treatable, and thus, should be assessed and diagnosed prior to the age of 18 in order to provide the best possible outcome later in life. As a consequence, patients as well as clinicians may be cautiously optimistic about the prognosis of a PD. In addition, if PDs and PD symptoms are not as stable as previously thought, this raises the question whether it is still appropriate to consider stability as a central feature of PDs? In other words, is it still reasonable to refer to a PD or PD symptoms, if the concept itself depends on numerous conceptual, methodological, genetic, and environmental factors? Or is it rather the general level of personality functioning (i.e., self and interpersonal functioning), which is conceptually separated from PD categories and symptoms, that actually determines a PD? This issue, in turn, emphasizes the current shift to more dimensional conceptualizations, as defined in the AMPD or ICD-11. In fact, both models introduce a radical change in the structure and diagnosis of PDs, by conceptualizing PDs as core impairments in self-and interpersonal functioning, amplified by a severity ranking and specific trait specifiers related to negative affectivity, detachment, dissociality (i.e., antagonism in the AMPD), disinhibition, and/or anankastia in the ICD-11 and psychoticism in the AMPD. We suggest that moving away from PD categories and PD symptoms helps clinicians to perceive the patient as a whole, by refocusing on the original meaning of personality, that is the subjective experience of what it means to be human (71). This may help to not only see if patients suffer, but also how they suffer. While the classification of severity may help inform clinical prognosis and intensity of treatment, the classification of trait specifiers may help to identify individual problems, resulting in more individualized, tailor-made treatments (72, 73).

To this date, the literature currently lacks data about the stability of the general level of personality functioning. Although we have reasons to think that it may be more stable, e.g., (12, 13), this remains to be proven. We therefore suggest that future research should focus more intensively on personality functioning and specific trait expressions in order to determine whether AMPD’s and ICD-11’s new conceptualizations clarify the issue of stability over time. Specifically, studies should investigate the course and outcome of personality functioning and pathological personality traits from childhood to late adulthood. Thereby, research should increasingly rely on dimensional assessments and longer follow-up intervals. Future work on the etiological origins of these constructs and the mechanisms by which these constructs evolve over time, will be of great importance. Moreover, future research needs to address methodological factors to prevent unnuanced responses to the complex notion of stability. In fact, researchers still often use the general term “stability” without being explicit regarding the type of stability they are referring to. This is particularly problematic as different types of stability can vary substantially as pointed out in the present review. In addition, future studies should incorporate more sophisticated sampling and statistical procedures to overcome possible limitations. In particular, studies should focus on multi-wave study designs, including multiple measurement points, in order to analyze the shape of each person’s individual trajectory and distinguishing true change from measurement error (74). Furthermore, studies of high-risk samples, especially in childhood and adolescence, may be crucial as these children and adolescents are particularly at risk of developing maladaptive personality traits and PD prevalence rates among these samples are alarmingly high. Finally, and most importantly, upcoming research should address genetic, contextual, and situational factors that may influence the course of PDs or personality functioning over the lifespan. After all, while the direction of change is known, the causes of change remain unclear.

## 7. Conclusion

In recent decades, research on the stability of PDs has considerably increased, yet it remains a much-debated topic as it is foremost a conceptual and methodological endeavor. This narrative review, however, has highlighted key findings from the current literature, suggesting comparable stability estimates in adolescence and adulthood, with considerable improvement over time. Future work may, eventually, determine whether the new conceptualization will clarify some of the issues related to the stability of PDs. Nevertheless, it should be acknowledged that a symptomatic remission is not necessarily accompanied by a full recovery, with most PD patients never managing to fully participate in society, despite considerable remission. Understanding the process of change is thus particularly important, in order to identify protective factors, that potentially might mitigate long-term impairments. Taken together, these findings challenge our current understanding of PDs in terms of disorders and symptoms and argue instead in favor of the AMPD and ICD-11 reintroducing the idea of self and interpersonal functioning as the core feature of PDs. This might enable clinicians to perceive the patient as a whole, by identifying individual problems, which, could, ultimately, contribute to more personalized and tailor-made treatments.

## Author contributions

DH and BB contributed to conceiving and designing the present manuscript. DH and SS conducted the literature search. DH wrote the first draft of the manuscript. SS, DB, MB, CB, MS, KS, and BB commented on an earlier draft of this article and supervised the entire process. All authors contributed to the article and approved the submitted version.

## Funding

DH was funded by an individual PhD fellowship from the Fonds National de la Recherche du Luxembourg (FNR).

## Conflict of interest

The authors declare that the research was conducted in the absence of any commercial or financial relationships that could be construed as a potential conflict of interest.

The reviewer YC declared a past co-authorship with one of the authors BB to the handling editor.

## Publisher’s note

All claims expressed in this article are solely those of the authors and do not necessarily represent those of their affiliated organizations, or those of the publisher, the editors and the reviewers. Any product that may be evaluated in this article, or claim that may be made by its manufacturer, is not guaranteed or endorsed by the publisher.

## References

[1] Grilo C McGlashan T Oldham J. Course and stability of personality disorders. J Psychiatr Pract. (1998) 4:61–75. doi: 10.1097/00131746-199803000-00001

[2] Zanarini MC Frankenburg FR Reich DB Silk KR Hudson JI McSweeney LB. The subsyndromal phenomenology of borderline personality disorder: a 10-year follow-up study. Am J Psychiatry. (2007) 164:929–35. doi: 10.1176/ajp.2007.164.6.929, PMID: 17541053

[3] Shea MT Yen S. Stability as a distinction between axis I and axis II disorders. J Personal Disord. (2003) 17:373–86. doi: 10.1521/pedi.17.5.373.22973, PMID: 14632373

[4] Morey LC Hopwood CJ. Stability and change in personality disorders. Annu Rev Clin Psychol. (2013) 9:499–528. doi: 10.1146/annurev-clinpsy-050212-185637 23245342

[5] Hopwood CJ Bleidorn W. Stability and change in personality and personality disorders. Curr Opin Psychol. (2018) 21:6–10. doi: 10.1016/j.copsyc.2017.08.034 28923391

[6] American Psychiatric Association. Diagnostic and statistical manual of mental disorders. Washington, DC: American Psychiatric Association (2013).

[7] World Healt Organization. International statistical classification of diseases and related health problems. Available at: https://icd.who.int/browse10/2019/en#/. (Accessed November 15, 2022).

[8] Roche MJ. Examining the alternative model for personality disorder in daily life: evidence for incremental validity. Personal Disord. (2018) 9:574–83. doi: 10.1037/per0000295, PMID: 29927292

[9] Wright AGC Hopwood CJ Skodol AE Morey LC. Longitudinal validation of general and specific structural features of personality pathology. J Abnorm Psychol. (2016) 125:1120–34. doi: 10.1037/abn0000165, PMID: 27819472 PMC5119768

[10] d’Huart D Steppan M Seker S Bürgin D Boonmann C Birkhölzer M. Prevalence and 10-year stability of personality disorders from adolescence to young adulthood in a high-risk sample. Front Psychiatry. (2022) 13:13. doi: 10.3389/fpsyt.2022.840678 PMC8987201 35401274

[11] Landis JR Koch GG. The measurement of observer agreement for categorical data. Biometrics. (1977) 33:159. doi: 10.2307/2529310 843571

[12] Pearson KI. Mathematical contributions to the theory of evolution—VII. On the correlation of characters not quantitatively measurable. Phil Trans R Soc A. (1900) 195:1–47. doi: 10.1098/rsta.1900.0022

[13] Lenzenweger MF Johnson MD Willett JB. Individual growth curve analysis illuminates stability and change in personality disorder features: the longitudinal study of personality disorders. Arch Gen Psychiatry. (2004) 61:1015–24. doi: 10.1001/archpsyc.61.10.1015, PMID: 15466675

[14] Muthén B Shedden K. Finite mixture modeling with mixture outcomes using the EM algorithm. Biometrics. (1999) 55:463–9. doi: 10.1111/j.0006-341X.1999.00463.x, PMID: 11318201

[15] Moher D Shamseer L Clarke M Ghersi D Liberati A Petticrew M. Preferred reporting items for systematic review and meta- analysis protocols (PRISMA-P) 2015 statement. Systematic Reviews. (2015). 4:1–9. doi: 10.1186/2046-4053-4-1, PMID: 25554246 PMC4320440

[16] Stroup D Berlin J. A Morton S. C Olkin I Williamson G. D Rennie D. Meta-analysis of observational studies in epidemiology: a proposal for reporting. Meta-analysis of Observational Studies in Epidemiology (MOOSE) group. JAMA. 283:2008–2012. doi: 10.1001/jama.283.15.2008, PMID: 10789670

[17] Crick NR Murray-Close D Woods K. Borderline personality features in childhood: a short-term longitudinal study. Dev Psychopathol. (2005) 17:1051–70. doi: 10.1017/S0954579405050492, PMID: 16613430

[18] de Clercq B van Leeuwen K van den Noortgate W de Bolle M de Fruyt F. Childhood personality pathology: dimensional stability and change. Dev Psychopathol. (2009) 21:853–69. doi: 10.1017/S0954579409000467, PMID: 19583887

[19] Bernstein DP Cohen P Velez CN Schwab-Stone M Siever LJ Shinsato L. Prevalence and stability of the DSM-III-R personality disorders in a community-based survey of adolescents. Am J Psychiatry. (1993) 150:1237–43. PMID: 8328570 10.1176/ajp.150.8.1237

[20] Bornovalova MA Hicks BM Iacono WG McGue M. Stability, change, and heritability of borderline personality disorder traits from adolescence to adulthood: a longitudinal twin study. Dev Psychopathol. (2009) 21:1335–53. doi: 10.1017/S0954579409990186, PMID: 19825271 PMC2789483

[21] Chanen AM Jackson HJ McGorry PD Allot KA Clarkson V Hok PY. Two-year stability of personality disorder in older adolescent outpatients. J Personal Disord. (2004) 18:526–41. doi: 10.1521/pedi.18.6.526.54798, PMID: 15615665

[22] Greenfield B Henry M Lis E Slatkoff J Guile JM Dougherty G. Correlates, stability and predictors of borderline personality disorder among previously suicidal youth. Eur Child Adolesc Psychiatry. (2015) 24:397–406. doi: 10.1007/s00787-014-0589-9, PMID: 25084977

[23] Grilo CM Becker DF Edell WS McGlashan TH. Stability and change of DSM-III-R personality disorder dimensions in adolescents followed up 2 years after psychiatric hospitalization. Compr Psychiatry. (2001) 42:364–8. doi: 10.1053/comp.2001.26274, PMID: 11559862

[24] Hamlat EJ Hankin BL Young JF. Developmental course of personality disorder traits in childhood and adolescence. J Personal Disord. (2020) 34:25–43. doi: 10.1521/pedi_2019_33_433, PMID: 31084556 PMC6980182

[25] Johnson JG Cohen P Kasen S Skodol AE Hamagami F Brook JS. Age-related change in personality disorder trait levels between early adolescence and adulthood: a community-based longitudinal investigation. Acta Psychiatr Scand. (2000) 102:265–75. doi: 10.1034/j.1600-0447.2000.102004265.x, PMID: 11089726

[26] Strandholm T Kiviruusu O Karlsson L Pankakoski M Pelkonen M Marttunen M. Stability and change in personality disorder symptoms in 1-year follow-up of depressed adolescent outpatients. J Nerv Ment Dis. (2017) 205:15–22. doi: 10.1097/NMD.0000000000000623, PMID: 27922907

[27] Yen S Gagnon K Spirito A. Borderline personality disorder in suicidal adolescents. Personal Ment Health. (2013) 7:89–101. doi: 10.1002/pmh.1216, PMID: 24343935 PMC4414329

[28] Alvarez-Tomàs I Soler J Bados A Martin-Blanco A Elices M Carmona C. Long-term course of borderline personality disorder: a prospective 10-year follow-up study. J Personal Disord. (2017) 31:590–605. doi: 10.1521/pedi_2016_30_269, PMID: 27749187

[29] Black DW Baumgard CH Bell SE. A 16-to 45-year follow-up of 71 men with antisocial personality disorder. Compr Psychiatry. (1995) 36:130–40. doi: 10.1016/S0010-440X(95)90108-6, PMID: 7758299

[30] Bukh JD Bech P Kessing LV. Diagnostic stability of comorbid personality disorders among patients fully or partially remitted from first-episode depression: a 5-year follow-up study. J Personal Disord. (2017) 31:208–20. doi: 10.1521/pedi_2016_30_253, PMID: 27322576

[31] Conway CC Boudreaux M Oltmanns TF. Dynamic associations between borderline personality disorder and stressful life events over five years in older adults. Personal Disord. (2018) 9:521–9. doi: 10.1037/per0000281, PMID: 29461847 PMC6098988

[32] de Groot MH Franken IH van der Meer CW Hendriks VM. Stability and change in dimensional ratings of personality disorders in drug abuse patients during treatment. J Subst Abus Treat. (2003) 24:115–20. doi: 10.1016/S0740-5472(02)00351-3, PMID: 12745028

[33] Durbin CE Klein DN. Ten-year stability of personality disorders among outpatients with mood disorders. J Abnorm Psychol. (2006) 115:75–84. doi: 10.1037/0021-843X.115.1.75, PMID: 16492098

[34] Farabaugh A Mischoulon D Schwartz F Pender M Fava M Alpert J. Dysfunctional attitudes and personality disorder comorbidity during long-term treatment of MDD. Depress Anxiety. (2007) 24:433–9. doi: 10.1002/da.20174, PMID: 17086540

[35] Hopwood CJ Morey LC Donnellan MB Samuel DB Grilo CM McGlashan TH. Ten-year rank-order stability of personality traits and disorders in a clinical sample. J Pers. (2013) 81:335–44. doi: 10.1111/j.1467-6494.2012.00801.x 22812532 PMC3593979

[36] Kullgren G Armelius BA. The concept of personality organization: a long-term comparative follow-up study with special reference to borderline personality organization. J Personal Disord. (1990) 4:203–12. doi: 10.1521/pedi.1990.4.2.203

[37] Laptook R. Ten-year stability of depressive personality disorder in depressed outpatients. Am J Psychiatry. (2006) 163:865–71. doi: 10.1176/ajp.2006.163.5.865, PMID: 16648328

[38] Lenzenweger MF. Stability and change in personality disorder features: the longitudinal study of personality disorders. Arch Gen Psychiatry. (1999) 56:1009–15. doi: 10.1001/archpsyc.56.11.1009 10565501

[39] Lopez-Castroman J Galfalvy H Currier D Stanley B Blasco-Fontecilla H Baca-Garcia E. Personality disorder assessments in acute depressive episodes: stability at follow-up. J Nerv Ment Dis. (2012) 200:526–30. doi: 10.1097/NMD.0b013e318257c6ab, PMID: 22652618 PMC3785086

[40] Loranger AW Lenzenweger MF Gartner AF Susman VL Herzig J Zammit GK. Trait-state artifacts and the diagnosis of personality-disorders. Arch Gen Psychiatry. (1991) 48:720–8. doi: 10.1001/archpsyc.1991.01810320044007, PMID: 1883255

[41] Mulder RT Joyce PR Frampton CMA. Personality disorders improve in patients treated for major depression. Acta Psychiatr Scand. (2010) 122:219–25. doi: 10.1111/j.1600-0447.2009.01502.x, PMID: 19895619

[42] Nestadt G Di C Samuels JF Bienvenu OJ Reti IM Costa P. The stability of DSM personality disorders over twelve to eighteen years. J Psychiatr Res. (2010) 44:1–7. doi: 10.1016/j.jpsychires.2009.06.009, PMID: 19656527 PMC2813415

[43] Nysaeter TE Nordahl HM. Comorbidity of borderline personality disorder with other personality disorders in psychiatric outpatients: how does it look at 2-year follow-up? Nord J Psychiatry. (2012) 66:209–14. doi: 10.3109/08039488.2011.621976, PMID: 22017242

[44] Paris J Zweig-Frank H. A 27-year follow-up of patients with borderline personality disorder. Compr Psychiatry. (2001) 42:482–7. doi: 10.1053/comp.2001.26271, PMID: 11704940

[45] Reichborn-Kjennerud T Czajkowski N Ystrom E Orstavik R Aggen SH Tambs K. A longitudinal twin study of borderline and antisocial personality disorder traits in early to middle adulthood. Psychol Med. (2015) 45:3121–31. doi: 10.1017/S0033291715001117, PMID: 26050739 PMC4589465

[46] Riihimaki K Vuorilehto M Isometsa E. Borderline personality disorder among primary care depressive patients: a five-year study. J Affect Disord. (2014) 155:303–6. doi: 10.1016/j.jad.2013.10.050, PMID: 24268615

[47] Schilders MR Ogloff JRP. Stability of life-time psychiatric diagnoses among offenders in community and prison settings. J Forensic Psychiatry Psychol. (2017) 28:133–54. doi: 10.1080/14789949.2016.1263676

[48] Silk KR Lohr NE Ogata SN Westen D. Borderline inpatients with affective disorder: preliminary follow-up data. J Personal Disord. (1990) 4:213–24. doi: 10.1521/pedi.1990.4.2.213

[49] Trull TJ Goodwin AH. Relationship between mood changes and the report of personality-disorder symptoms. J Pers Assess. (1993) 61:99–111. doi: 10.1207/s15327752jpa6101_7 8377105

[50] Vaglum P Friis S Karterud S Mehlum L Vaglum S. Stability of of severe personality disorder diagnosis: a 2-to 5-year prospective study. J Personal Disord. (1993) 7:348–53. doi: 10.1521/pedi.1993.7.4.348

[51] Vater A Ritter K Strunz S Ronningstam EF Renneberg B Roepke S. Stability of narcissistic personality disorder: tracking categorical and dimensional rating systems over a two-year period. Personal Disord. (2014) 5:305–13. doi: 10.1037/per0000058 24512458

[52] Vergara-Moragues E Gonzalez-Saiz F Lozano OM Garcia AV. Psychopathological stability of personality disorders in substance abuse patients treated in a therapeutic community. J Addict Dis. (2013) 32:343–53. doi: 10.1080/10550887.2013.854154, PMID: 24325768

[53] Vrabel KR Ro O Martinsen EW Hoffart A Rosenvinge JH. Five-year prospective study of personality disorders in adults with longstanding eating disorders. Int J Eat Disord. (2010) 43:22–8. doi: 10.1002/eat.20662, PMID: 19247987

[54] Zanarini MC Frankenburg FR Reich DB Garrett F. Time to attainment of recovery from borderline personality disorder and stability of recovery: a 10-year prospective follow-up study. Am J Psychiatry. (2010) 167:663–7. doi: 10.1176/appi.ajp.2009.09081130, PMID: 20395399 PMC3203735

[55] Zanarini MC Frankenburg FR Reich B Fitzmaurice G. Attainment and stability of sustained symptomatic remission and recovery among patients with borderline personality disorder and axis II comparison subjects: a 16-year prospective follow-up study. Am J Psychiatry. (2012) 169:476–83. doi: 10.1176/appi.ajp.2011.11101550, PMID: 22737693 PMC3509999

[56] Chanen AM McCutcheon LK. Complex case: personality disorder in adolescence: the diagnosis that dare not speak its name. Personal Ment Health. (2008) 2:35–41. doi: 10.1002/pmh.28

[57] Miller AL Muehlenkamp JJ Jacobson CM. Fact or fiction: diagnosing borderline personality disorder in adolescents. Clin Psychol Rev. (2008) 28:969–81. doi: 10.1016/j.cpr.2008.02.004 18358579

[58] Shiner RL Allen TA. Assessing personality disorders in adolescents: seven guiding principles. Clin Psychol Sci Pract. (2013) 20:361–77. doi: 10.1111/cpsp.12047

[59] Chanen A Sharp C Hoffman P Global Alliance for Prevention and Early Intervention for Borderline Personality Disorder. Prevention and early intervention for borderline personality disorder: Anovel public health priority. World Psychiatry. (2017) 16:215–6. doi: 10.1002/wps.20429, PMID: 28498598 PMC5428197

[60] Fossati A Somma A. The assessment of personality pathology in adolescence from the perspective of the alternative DSM-5 model for personality disorder. Curr Opin Psychol. (2021) 37:39–43. doi: 10.1016/j.copsyc.2020.07.015, PMID: 32827876

[61] Chanen AM Thompson KN. The age of onset of personality disorders. Age of onset of mental disorders: Springer; (2019) 183–201.

[62] Balsis S Segal DL Donahue C. Revising the personality disorder diagnostic criteria for the diagnostic and statistical manual of mental disorders-fifth edition (DSM-V): consider the later life context. Am J Orthopsychiatry. (2009) 79:452–60. doi: 10.1037/a0016508, PMID: 20099936

[63] Newton-Howes G Clark LA Chanen A. Personality disorder across the life course. Lancet. (2015) 385:727–34. doi: 10.1016/S0140-6736(14)61283-6 25706218

[64] Roberts BW Walton KE Viechtbauer W. Patterns of mean-level change in personality traits across the life course: a meta-analysis of longitudinal studies. Psychol Bull. (2006) 132:1–25. doi: 10.1037/0033-2909.132.1.1, PMID: 16435954

[65] Wright AG Pincus AL Lenzenweger MF. Interpersonal development, stability, and change in early adulthood. J Pers. (2012) 80:1339–72. doi: 10.1111/j.1467-6494.2012.00761.x, PMID: 22224462 PMC3524989

[66] Grilo CM Sanislow CA Gunderson JG Pagano ME Yen S Zanarini MC. Two-year stability and change of schizotypal, borderline, avoidant, and obsessive-compulsive personality disorders. J Consult Clin Psychol. (2004) 72:767–75. doi: 10.1037/0022-006X.72.5.767, PMID: 15482035 PMC3289406

[67] Samuel DB Hopwood CJ Ansell EB Morey LC Sanislow CA Markowitz JC. Comparing the temporal stability of self-report and interview assessed personality disorder. J Abnorm Psychol. (2011) 120:670–80. doi: 10.1037/a0022647, PMID: 21443287 PMC4793384

[68] Gunderson JG Shea MT Skodol AE McGlashan TH Morey LC Stout RL. The collaborative longitudinal personality disorders study: development, aims, design, and sample characteristics. J Personal Disord. (2000) 14:300–15. doi: 10.1521/pedi.2000.14.4.300, PMID: 11213788

[69] Zanarini MC Frankenburg FR Hennen J Reich DB Silk KR. The McLean study of adult development (MSAD): overview and implications of the first six years of prospective follow-up. J Personal Disord. (2005) 19:505–23. doi: 10.1521/pedi.2005.19.5.505, PMID: 16274279

[70] Zanarini MC Frankenburg FR Hennen J Silk KR. The longitudinal course of borderline psychopathology: 6-year prospective follow-up of the phenomenology of borderline personality disorder. Am J Psychiatry. (2003) 160:274–83. doi: 10.1176/appi.ajp.160.2.274, PMID: 12562573

[71] Sharp C Wall K. DSM-5 level of personality functioning: refocusing personality disorder on what it means to be human. Annu Rev Clin Psychol. (2021) 17:313–37. doi: 10.1146/annurev-clinpsy-081219-105402, PMID: 33306924

[72] Bach B Markon K Simonsen E Krueger RF. Clinical utility of the DSM-5 alternative model of personality disorders: six cases from practice. J Psychiatr Pract. (2015) 21:3–25. doi: 10.1097/01.pra.0000460618.02805.ef 25603448

[73] Bach B First MB. Application of the ICD-11 classification of personality disorders. BMC Psychiatry. (2018) 18:1–14. doi: 10.1186/s12888-018-1908-3 30373564 PMC6206910

[74] Singer J Willet J. A framework for investigating change over time. Applied longitudinal data analysis: modeling change and event occurrence Oxford University Press: USA (2003); 315: 115–139.

